# Effect of antiresorptive and anabolic bone therapy on development of osteoarthritis in a posttraumatic rat model of OA

**DOI:** 10.1186/s13075-015-0829-5

**Published:** 2015-11-06

**Authors:** Cedo M. Bagi, Edwin Berryman, David E. Zakur, Dean Wilkie, Catharine J. Andresen

**Affiliations:** Comparative Medicine, Global Science and Technology, Pfizer Global Research and Development, Pfizer Inc., 100 Eastern Point Road, Groton, CT 06340 USA; Investigative Pathology, Drug Safety Research and Development, Pfizer Inc., 100 Eastern Point Road, Groton, CT 06340 USA; Global Science and Technology, Pfizer Global Research and Development, Pfizer Inc., 100 Eastern Point Road, Groton, CT 06340 USA

**Keywords:** Osteoarthritis, Zoledronic acid, Parathyroid hormone, Dynamic weight bearing, Contrast-enhanced μCT, Histology, Dynamic histomorphometry

## Abstract

**Introduction:**

Osteoarthritis (OA) is a leading cause of disability, but despite the high unmet clinical need and extensive research seeking dependable therapeutic interventions, no proven disease-modifying treatment for OA is currently available. Due to the close interaction and interplay between the articular cartilage and the subchondral bone plate, it has been hypothesized that antiresorptive drugs can also reduce cartilage degradation, inhibit excessive turnover of the subchondral bone plate, prevent osteophyte formation, and/or that bone anabolic drugs might also stimulate cartilage synthesis by chondrocytes and preserve cartilage integrity. The benefit of intensive zoledronate (Zol) and parathyroid hormone (PTH) therapy for bone and cartilage metabolism was evaluated in a rat model of OA.

**Methods:**

Medial meniscectomy (MM) was used to induce OA in male Lewis rats. Therapy with Zol and human PTH was initiated immediately after surgery. A dynamic weight-bearing (DWB) system was deployed to evaluate the weight-bearing capacity of the front and hind legs. At the end of the 10-week study, the rats were euthanized and the cartilage pathology was evaluated by contrast (Hexabrix)-enhanced μCT imaging and traditional histology. Bone tissue was evaluated at the tibial metaphysis and epiphysis, including the subchondral bone. Histological techniques and dynamic histomorphometry were used to evaluate cartilage morphology and bone mineralization.

**Results:**

The results of this study highlight the complex changes in bone metabolism in different bone compartments influenced by local factors, including inflammation, pain and mechanical loads. Surgery caused severe and extensive deterioration of the articular cartilage at the medial tibial plateau, as evidenced by contrast-enhanced μCT and histology. The study results showed the negative impact of MM surgery on the weight-bearing capacity of the operated limb, which was not corrected by treatment. Although both Zol and PTH improved subchondral bone mass and Zol reduced serum CTX-II level, both treatments failed to prevent or correct cartilage deterioration, osteophyte formation and mechanical incapacity.

**Conclusions:**

The various methods utilized in this study showed that aggressive treatment with Zol and PTH did not have the capacity to prevent or correct the deterioration of the hyaline cartilage, thickening of the subchondral bone plate, osteophyte formation or the mechanical incapacity of the osteoarthritic knee.

## Introduction

Osteoarthritis (OA) is a complex disease of the entire joint, affecting the cartilage, bone and synovial membranes [1]. With the aging of the population in the United States, OA, as the most prevalent form of arthritis, contributes considerably to the economic and social burden on the health care system [2]. The multifactorial etiology of OA and the inevitable presence of risk factors such as abnormal loading, obesity, repetitive injuries and aging, which persist throughout the life cycle of the disease, make treatment extremely difficult [3]. Clearly, multitherapeutic approaches over longer durations will be necessary to effectively treat this debilitating disease. Despite the high unmet need and extensive research seeking dependable therapeutic interventions, no proven disease-modifying treatments for OA are currently available.

The mechanical and metabolic roles of the subchondral bone are considered critical for the health of the articular cartilage and for normal joint physiology [4, 5]. Increased turnover, osteophyte formation and osteosclerosis are the most common features of subchondral bone pathophysiology that frequently accompany cartilage degradation in OA patients [5, 6]. Interdependence of articular cartilage and subchondral bone, in both healthy and diseased states, led to suggestions to expand the use of drugs approved to treat osteoporosis (OP) to also treat bone and, subsequently, cartilage in OA patients [7–9]. This proposal may sound controversial since the previous studies seem to suggest that OA is inversely related to OP and that OA is associated with increased bone mineral density [10, 11]. However, proposals to use antiresorptive bone therapy to treat OA is largely based on the premise that this therapy will reduce cartilage degradation, inhibit excessive turnover of the subchondral bone plate and prevent bone loss in the limb affected by OA [6, 12]. Treatment with bisphosphonates has been shown to reduce cartilage degradation [13–15], have no effect [16, 17], or even to have a negative effect [18] on articular cartilage in animal models of OA. Clinical results, however, failed to demonstrate lessening of symptoms or improvement of radiographic progression in patients with OA treated with antiresorptive agents [19, 20]. Parathyroid hormone (PTH), known as teriparatide in drug form, has emerged as a major player in the maintenance and healing of bone, but the literature discussing usefulness of PTH to treat OA is less indicative [21, 22]. However, sharing of a mesenchymal cell lineage by osteoblasts and chondrocytes suggests that treatment with PTH might stimulate cartilage synthesis by chondrocytes and protect against cartilage degradation [23–27]. This implies that perhaps immediate and aggressive treatment with PTH will be more beneficial to prevent cartilage degeneration and initiate the healing in patients with posttraumatic OA (PTOA). Overall, published literature on bone therapies indicates that carefully selected antiresorptive and anabolic treatment could provide benefit to a selected patient population with OA. The data also suggest that aggressive preemptive antiresorptive therapy might be more effective in inhibiting cartilage degradation and excessive turnover of the subchondral bone in posttraumatic states [15, 28, 29]. Also, it should be noted that the majority of preclinical studies utilize intermittent dosing with PTH based on bone-efficacy data [30], even though the work done by Kudo and colleagues showed similar efficacy on cartilage repair with continuous and intermittent administration of PTH [26].

Damage to the meniscus or ligaments within the knee caused by trauma or repeated microtrauma are widely accepted as common causes of PTOA in this joint [27]. A unilateral medial meniscectomy (MM) in rats primarily results in fast and progressive degenerative changes of the articular cartilage of the medial tibial plateau, whereas subchondral bone pathology (sclerosis and formation of osteophytes) is deemed secondary and reflects local adaptation of the skeleton to new mechanical demands [31–34]. Patients with acute traumatic cartilage injury will probably benefit the most from prompt and aggressive therapy that can minimize cartilage damage, initiate cartilage repair, and prevent secondary changes of the subchondral bone.

This manuscript reports the outcomes of surgically imposed meniscal injury on cartilage health and the load-bearing capacity of the operated limb, and the reaction of hyaline cartilage and bone to intensive treatment with antiresorptive (zoledronate) and anabolic bone (PTH) therapy. Cartilage and bone pathology was evaluated by using serum biomarkers of bone and cartilage metabolism, micro-computed tomography (μCT) analysis of cartilage and bone tissue and cartilage histology.

## Methods

### Animals and management

Four-month-old male Lewis rats (Charles River Laboratories, Portage, MI, USA) weighing approximately 350 g at the beginning of the experiments were used in this study. All in vivo procedures were approved by the Institutional Animal Care and Use Committee (IACUC) at Pfizer (Andover, MA, USA) and were performed in accordance with the Guide for the Care and Use of Laboratory Animals and with the US National Institutes of Health (NIH) Publication No. 85-23, revised in 1996 [35]. All efforts were made to minimize animal pain and distress and to minimize the number of animals used. The rats were pair housed in ventilated Innovive cages in a temperature- and humidity-controlled room on a regular 12-hour light/dark cycle. Irradiated LabDiet™ 5053 (Purina, Richmond, IN, USA) and water were provided ad libitum. The rats were acclimated for 1 week prior to use in the study. A total of 48 rats were used for the 10-week study, with 12 rats per group. The rats were randomized to four study groups based on their body weights on the day before surgery. A group of 12 rats received sham surgery, and another group of 36 rats underwent MM surgery. The four study groups were as follows: sham control group (Sham), MM vehicle-treated group (MM + veh), MM + zoledronate group (MM + Zol) and MM + PTH group (MM + PTH).

### Surgery

The rats were induced and maintained under anesthesia using isoflurane. One dose of carprofen (Pfizer Animal Health, New York, NY, USA) and sustained-release buprenorphine (Zoopharm, Windsor, CO, USA) were administered prior to surgery for analgesic coverage. The right knee was shaved, aseptically prepared, and draped for surgery. In the sham group, a surgical approach to the medial collateral ligament (MCL) on the right hind limb was completed by only cutting the skin while the MCL was left intact. The surgical incision was closed in two layers using absorbable sutures. In the surgery groups, medial meniscectomy (MM) was performed by fully transecting both the MCL and the medial meniscus of the right hind limb, followed by closure in two layers using absorbable sutures [36]. All of the rats were euthanized at 10 weeks post surgery.

### Dosing and bone labeling

The rats in the Sham and MM + veh control groups (groups 1 and 2) received vehicle (sterile water used for injections) at 1 mL/kg subcutaneously (sc) 5 days/week, starting on the day of surgery. The rats in group 3 received zoledronic acid (Zol; Sargent Pharmaceuticals 25021-801, Schaumburg, IL, USA) at 100 μg/kg, sc, twice/week [18], and the rats in group 4 received human PTH (hPTH 1-34; Sigma-Aldrich P3796, St. Louis, MO, USA) at 40 μg/kg, sc, five times/week [25], starting on the day of surgery. To label actively mineralized bone surfaces, all of the rats in the study received calcein (Sigma-Aldrich Cat. No. C-0875, St. Louis, MO, USA) at 10 mg/kg (3.3 mL/kg) 13 days before necropsy and alizarin (Sigma-Aldrich Cat. No. A-5533, St. Louis, MO, USA) at 30 mg/kg (3.0 mL/kg) 3 days before necropsy.

### Body weight, sample collection and serum analyses

Body weight was recorded twice weekly throughout the study. At the end of the 10-week study the entire right hind limb was carefully harvested, skinned, and cleaned of the soft tissue, with care taken not to disrupt the knee joint. The limbs were wrapped in saline-soaked gauze and frozen at -20 °C for ex vivo imaging and histological analyses of the tibial articular cartilage and bone. Blood was collected by jugular venipuncture under isoflurane anesthesia. Serum was analyzed for biomarkers of bone formation and bone and cartilage degradation at 5 and 10 weeks post surgery. Osteocalcin was assessed by rat EIA kit (Cat. No. BT-490, Biomedical Technologies, Stoughton, MA, USA) and procollagen type 1 N-terminal propeptide (P1NP) was quantified in serum samples by the liquid chromatography/mass spectrometry (LC/MS) method [37]. Cross-linked type I collagen C-terminal telopeptide (CTX-I) was analyzed by rat LAPS™ Assay (Cat. No. AC-06 F1) and C-terminal telopeptide of type II collagen (CTX-II) by CartiLaps™ Assay (Cat. No. AC08F1) all produced by Immunodiagnostics Systems, Scottsdale, AZ, USA.

At the end of the study serum chemistry analysis included alanine aminotransferase (ALT, U/L), glutamic-oxaloacetic transaminase (AST, U/L), alkaline phosphatase (ALP, U/L), gamma-glutamyl transpeptidase (GGT, U/L), total bilirubin (TBIL, mg/dL), cholesterol (CHOL, mg/dL), glucose (GLUC, mg/dL), total protein (TP, g/dL), albumin (ALB, g/dL), globulin (BLOB, g/dL), albumin/globulin ratio (AG, ratio), blood urea nitrogen (BUN, mg/dL), creatinine (CREA, mg/dL), phosphorus (PHOS, mg/dL), calcium (Ca, mg/dL), sodium (Na, mmol/L), potassium (K, mmol/L) and chloride (Cl, mmol/L).

### Dynamic weight bearing

Dynamic weight-bearing (DWB) measurements were obtained before surgery, at week 5 and before euthanasia to assess the effects of surgery on the weight-bearing capacity of the hind and front legs. The DWB system (model BIO-SWB-R, Bioseb, Boulogne, France) is a noninvasive method used to obtain the weight and surface area of all four feet in a freely moving animal [38]. The system consists of a Plexiglas enclosure (22 × 22 × 30 cm) with a floor sensor consisting of an array of 44 × 44 sensors. A camera is affixed to the side of the enclosure to align the sensor directionally during the analysis phase. Raw pressure and live video recordings are transmitted to a tablet PC via a USB interface at a sampling frequency of 10 Hz. The data were analyzed using the DWB software, version 1.3. Zone parameters were set for the analysis as follows: ≥ 4 g for one sensor or a minimum of three adjacent sensors ≥ 2 g (to be considered a valid zone). For each time segment that was stable for more than 1 second, zones that met the above criteria were validated and assigned as either right or left and front or rear. Mean values for the weight and area of each zone were calculated over the entire testing period based on the length of time for each validated segment. For each testing period, the animals were placed into the chamber and allowed 20–30 seconds to explore prior to the data collection time of 3 minutes. The operator manually “validated” each test period by ensuring that each paw print corresponded to the appropriate paw, using the video footage as a reference. The following parameters were measured for each leg separately and for both the front and hind legs combined: body weight (g), weight put on the limb (g), percentage of weight placed on the limb and surface area of each paw (mm^2^).

### Radiology

All of the bone samples were X-rayed with the Faxitron Model MX20 specimen scanner (Faxitron Bioptics LLC., Tucson, AZ, USA) using the settings recommended by the manufacturer; exposure time 12–18 seconds at 31–35 kV. All of the samples were imaged at 3× magnification and were positioned horizontally with the center of the beam at the knee joint. Both frontal and lateral views of each sample were obtained. Radiographic images were used to assess the gross anatomy of the region of interest to be evaluated by μCT and to inspect the bone samples for the presence of fractures or other bone abnormalities.

### μCT and EPIC μCT measurements

μCT was conducted on the right knee joint, utilizing a MicroCT 100® computed tomography system (Scanco Medical, Bassersdorf, Switzerland) to obtain a scout three-dimensional image of the knee and ensure that the samples were reproducibly scanned and analyzed exactly at the same region of interest (ROI) in each specimen and that the size of the ROI that we selected allowed for meaningful analysis of bone structures at the proximal tibial epiphysis and metaphysis.

Following imaging of the entire knee, the femur and tibia were carefully separated to ensure that the articular cartilage and meniscus of the joint were not disrupted. The tibia was then cut above the tibiofibular junction and the proximal tibia was placed in a plastic custom-made positioning device to ensure consistent scanning. Precontrast scans of all the tibias were obtained using the MicroCT 100**®** with the following parameters: 800 slices, a 10-μm resolution, a total scanned area of 8.0 mm^2^, and source energy of 70 kVp, 115 μA at 8 W to capture the entire proximal tibia section.

Following precontrast μCT scans, 1.2 mL of Hexabrix (ioxaglate meglumine 39.3 % and ioxaglate sodium 19.6 %; Mallinckrodt, St. Louis, MO, USA) was added to a 15 mL conical tube and diluted with 1.8 mL of 0.1 M phosphate-buffered saline containing protease inhibitors (1 % Protease Inhibitor Cocktail Set I, CalBiochem, San Diego, CA, USA), yielding a 40 % solution of Hexabrix [39]. The tibia was then placed in this Hexabrix solution and was capped and incubated in a covered, rocking water bath at 37 °C for 3 hours. After the incubation period, the sample was removed, patted dry and placed in the plastic positioning device within a μCT holder containing a small amount of saline to help maintain sample hydration during scanning. Postsoak scanning of the right tibia was performed in the same manner as described above, except that the parameters were set differently to better visualize the cartilage, with source energy of 55 kVp, 145 μA at 8 W and an average scan time of 42 minutes per sample [39, 40].

### μCT evaluation of the cancellous bone at proximal tibial metaphysis

**Fig. 1:**
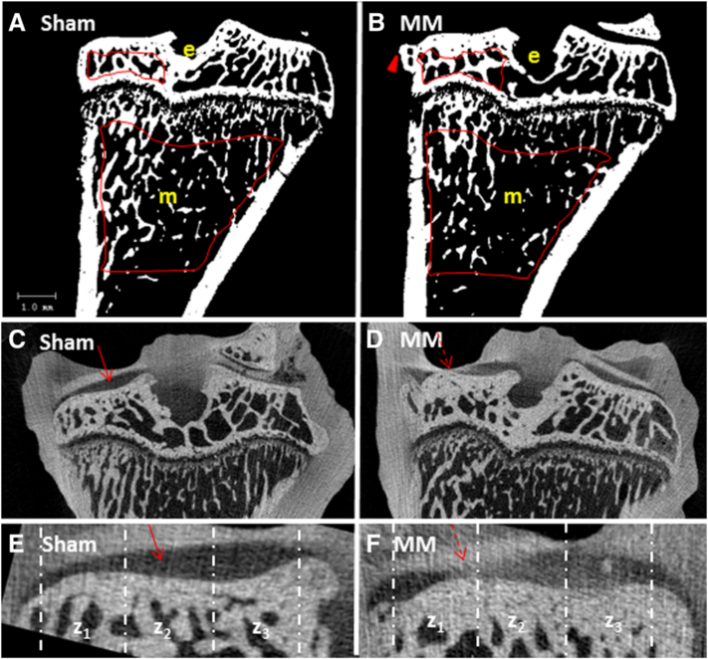
**a** and **b** show two-dimensional micro-computed tomography (μCT) images of the proximal tibia from sham control (Sham) (**a**) and medial meniscectomy vehicle-treated (MM + veh) rats (**b**). *Red line* indicated the area of cancellous bone evaluation at tibial epiphysis (e) and metaphysis (m). *Arrowhead* points at the osteophyte. **c** and **d** show EPIC μCT images of the same tibias depicted in (**a**) and (**b**). The *red arrow* indicates normal articular cartilage whereas the *dotted arrow* indicates osteoarthritic cartilage. **e** and **f** show larger EPIC μCT images of the articular cartilage. The length of the medial tibial plateau was measured for each sample and then divided into three zones ranging from 0.8 to 1.0 mm in length. Zone 1 (Z1) was placed on the outside of the medial edge of the joint and Zone 3 (Z3) on the inside of the tibial plateau adjacent to the central collateral ligaments. Zones are delineated by *dotted lines*. The *red arrow* indicates normal articular cartilage whereas the *dotted arrow* indicates osteoarthritic cartilage

The cancellous bone compartment of the metaphysis was analyzed 1 mm below the growth plate and extending 3 mm distally to include the metaphyseal secondary spongiosa. Cancellous bone was evaluated in an ROI drawn on 100 consecutive slices, each 1.0 mm in thickness, which best represented the central segment of the tibia [41, 42]. Cancellous bone parameters included bone mineral density (grams of calcium/bone volume), tissue volume (bone and bone marrow), bone volume, bone volume/tissue volume ratio, bone surface, bone surface/bone volume, trabecular number, trabecular thickness, trabecular separation (distance between individual trabeculae), connectivity diameter (connection between individual trabeculae), and structural model index for shape (Fig. 1a and b).

### μCT evaluation of the cancellous bone at proximal tibial epiphysis

An ROI 2.0 mm × 0.5 mm was drawn on the precontrast images to include only cancellous subchondral bone underlying the articular cartilage. This region was drawn on the 100 consecutive slices (1.0 mm total thickness). Cancellous bone parameters included bone mineral density (grams of calcium/bone volume), tissue volume (bone and bone marrow), bone volume, bone volume/tissue volume ratio, bone surface, bone surface/bone volume, trabecular number, trabecular thickness, trabecular separation (distance between individual trabeculae), connectivity diameter (connection between individual trabeculae), and structural model index for shape (Fig. 1a and b).

### μCT evaluation of the epiphyseal cartilage

Using the postcontrast scans, contour lines were drawn around an ROI that included the cartilage overlying the medial tibial plateau. The ROI was purposely drawn to include a small amount of bone and soft tissue around the cartilage to ensure that an appropriate threshold was selected to segment the cartilage from bone tissue and soft tissue according to histographical analysis of the tissues. After the lower (70) and the upper (440) threshold was determined, the contour lines were manually drawn with semiautomatic contouring applied every 3–10 slices over a total of 300 slices (3 mm) to capture most of the articular surface (Fig. 1c and d). The three-dimensional morphology of the entire articular cartilage layer drawn was then visualized and quantified in terms of average cartilage thickness, volume and surface area using direct distance transformation algorithms [43, 44].

Other ROIs were drawn and analyzed on this central midpoint of the articular surface, corresponding to standard histological evaluation techniques for the articular cartilage [45]. The length of the medial articular cartilage was measured and divided into three zones of equal length as already described for the subchondral bone. The parameters of articular cartilage included cartilage volume and cartilage thickness (Fig. 1e and f).

### Histology

After the completion of EPIC μCT imaging of the articular cartilage, six tibias were randomly chosen and placed in 10 % neutral buffered formalin for 72 hours prior to demineralization for 8 days in Immunocal (Decal Chemical Corp., Tillman, NY, USA). The tibias were then processed in paraffin and were serially sectioned at approximately 200 μm intervals into 5 μm-thick sections for staining. The slides were stained with hematoxylin and eosin (H&E) and toluidine blue for general structural evaluation and with safranin O for the evaluation of cartilage damage. Thickness and degeneration of the articular cartilage at the medial tibial plateau were determined on three longitudinal sections of the proximal tibia using an ocular micrometer. Cartilage thickness was measured separately on each of three zones, as suggested in the literature [45–47]. The severity of OA lesions was graded on a scale adopted from Osteoarthritis Research Society International (OARSI) histopathology instructions [45]. Five histological measures used in this study included damage score, osteophyte size (μm), significant cartilage degeneration width (μm), cartilage thickness (μm) and cartilage matrix loss width (μm).

Undecalcified bones were embedded in methyl methacrylate and were cut into 8 μm-thick longitudinal sections using a polycut sliding microtome (Leica Biosystems, Nussloch, Germany) or into 20 μm-thick sections using a bone cutting and grinding system (Exakt Norderstedt, Germany). The unstained sections were used first to assess new bone formation at the medial tibial epiphysis, and these sections were subsequently stained according to Von Kossa’s method to quantify mineralization.

### Statistical analysis

Data are given as means ± standard deviations (SDs). Differences were tested for significance using three-factor repeated-measures analysis of variance (ANOVA) with interactions (Sigma Plot, version 12.2, Systat Software, Chicago, IL, USA). Post hoc comparisons of means with a Bonferroni correction for multiple comparisons were performed only when interaction effects were significant. *p* values less than 0.05 were considered statistically significant.

## Results

### Animals

All of the rats enrolled in the study showed similar increases of 15 % in body weight, regardless of treatment. All of the rats that received the MM surgery developed OA, as evidenced by μCT and histology. Surgery or treatment with Zol and PTH did not affect serum chemistry parameters (data not shown).

### Serum biomarkers of bone and cartilage metabolism

After 5 weeks of dosing the MM rats showed higher CTX-II (*p* < 0.05) values compared to sham controls. Zoledronate-treated rats exhibited lower osteocalcin (*p* < 0.01), P1NP (*p* < 0.05) and CTX-II (*p* < 0.01) relative to MM controls. On the other hand, rats treated with PTH exhibited higher osteocalcin (*p* < 0.01) and lower CTX-II (*p* < 0.05) values compared to MM control rats (Table 1).

**Table 1:** Shows the serum biomarkers of bone and cartilage metabolism at the end of 5 and 10 weeks of the study

			5 weeks		
Parameter	Unit	Sham	MM + veh	MM + Zol	MM + PTH
Osteocalcin	ng/mL	32.8 ± 5.0	32.1 ± 5.1	24.3 ± 2.1^**^	60.4 ± 16.9^**^
P1NP	ng/mL	277.2 ± 73.7	274.8 ± 44.2	243.8 ± 43.8	285.5 ± 44.1
CTX-I	ng/mL	22.5 ± 4.4	25.0 ± 5.0	28.9 ± 4.7	30.6 ± 6.3
CTX-II	ng/mL	30.5 ± 9.9	39.3 ± 8.7^a^	13.2 ± 8.5^**^	30.4 ± 4.0^*^
			10 weeks		
Osteocalcin	ng/mL	47.1 ± 11.5	38.1 ± 9.3	27.8 ± 11.5	30.7 ± 3.9
P1NP	ng/mL	146.0 ± 22.0	174.7 ± 26.1	134.8 ± 29.7	163.7 ± 26.8
CTX-I	ng/mL	36.7 ± 3.1	40.5 ± 7.9	49.3 ± 7.6	36.2 ± 4.1
CTX-II	ng/mL	14.4 ± 4.1	20.9 ± 5.6^a^	11.6 ± 3.2^*^	21.4 ± 5.3

*Sham*, sham control group, *MM + veh* medial meniscectomy (MM) vehicle-treated group, *MM + Zol* MM + zoledronate group, *MM + PTH* MM *+* parathyroid hormone group, *P1NP* procollagen type 1 N-terminal propeptide, *CTX-I* cross-linked type I collagen C-terminal telopeptide, *CTX-II* C-terminal telopeptide of type II collagen

^*^ *p* < 0.05 or ^**^ < 0.01 relative to MM + veh rats

^a^ *p* < 0.05 relative to sham rats

After 10 weeks of dosing, the MM rats showed higher CTX-II (*p* < 0.05) values compared to sham controls. The rats treated with Zol exhibited lower CTX-II values (*p* < 0.05) compared to the MM controls. There was no difference in serum biomarkers between PTH-treated rats and MM controls at the end of the10-week study (Table 1).

### Dynamic weight bearing

**Fig. 2:**
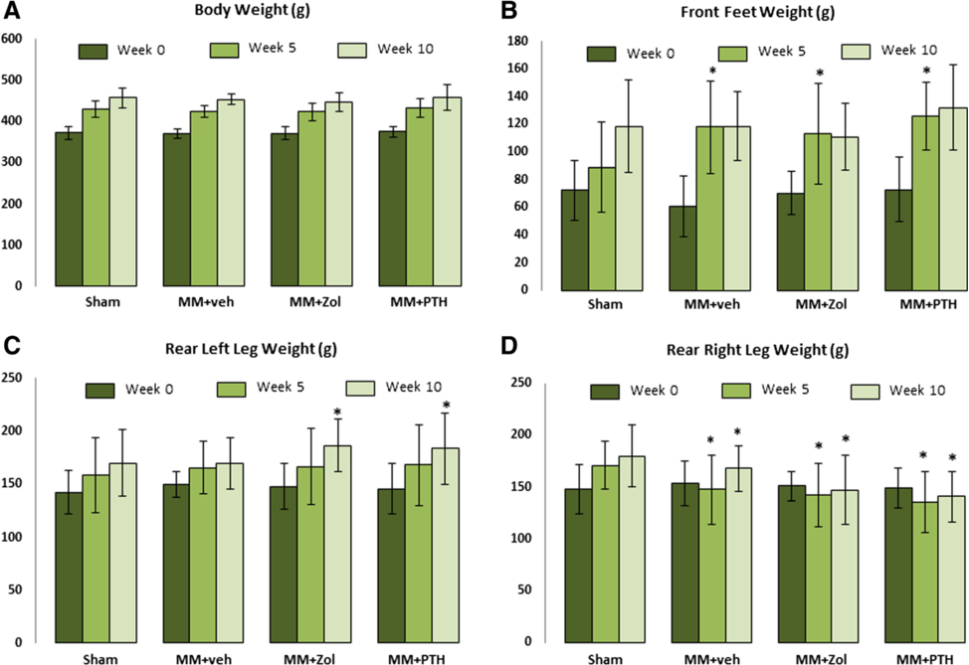
Changes in the body weight (**a**) and weight-bearing activity of the front feet (**b**), rear left leg (**c**) and on the operated rear right leg (**d**). The weight-bearing load on the front feet in all three groups of medial meniscectomy (MM) rats was greater at the 5-week time point compared to the sham control (Sham) rats. The load on the rear right leg was diminished in all three groups of MM rats at both week 5 and week 10. ^*^ *p* < 0.05 relative to Sham rats

During the 10 weeks of the study, the weight gain was similar in all four experimental groups; however, the MM rats showed a different pattern of weight distribution compared to the sham controls. The rats tended to shift their weight forward to their front legs gradually as they became heavier with age (Fig. 2). In all of the MM rats, the shift toward the front legs occurred earlier and was clearly visible at the 5-week time point; therefore, the weight-bearing load on the front feet in the MM rats was approximately 20 % greater than in the sham controls (Fig. 2). During the second half of the study, the sham control rats increased the weight bearing on the front feet by 34 %, whereas weight bearing in the MM rats did not change. The load placed on the rear right leg steadily increased in the sham control rats as they gained weight during the course of the study, but the MM rats did not change their load-bearing activity on the operated limb despite the gain in body weight being similar between the Sham and MM groups. The weight-bearing loads placed on the left hind leg were also increased in MM rats treated with Zol and PTH (Fig. 2).

### μCT evaluation of cancellous bone at the tibial metaphysis

**Fig. 3:**
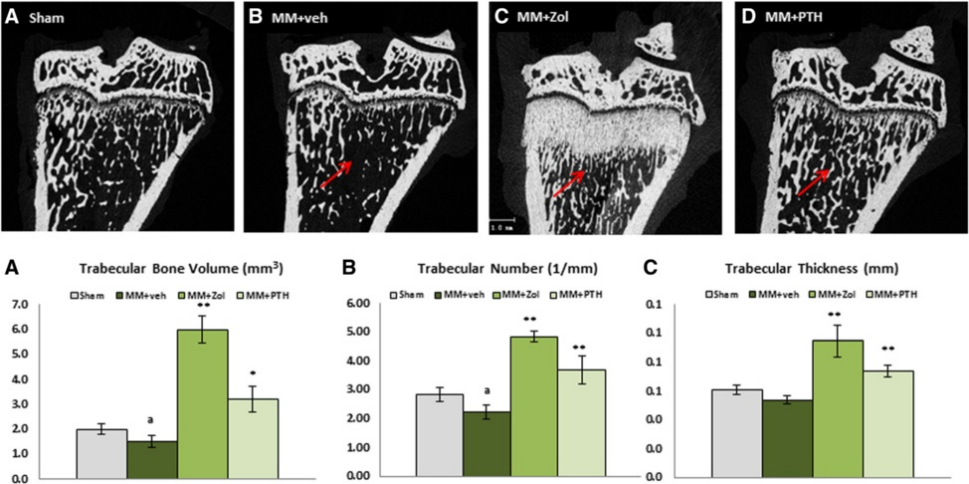
Two-dimensional micro-computed tomography (μCT) images of the proximal tibia from rats assign to study group 3A to 3D. *Arrows* indicate cancellous bone at the tibial metaphysis. Graph underneath depicts trabecular bone volume (**a**), trabecular number (**b**) and trabecular thickness (**c**) parameter obtained by μCT at proximal tibial metaphysis of the operated right leg. ^a^ *p* < 0.05 relative to sham control (Sham) rats; ^*^ *p* < 0.05 or ^**^ *p* < 0.01 relative to medial meniscectomy vehicle-treated (MM + veh) rats

Change in mechanical loading following the MM surgery resulted in significantly decreased bone volume and trabecular number in metaphyseal secondary spongiosa compared to the sham control rats. Treatment with Zol dramatically improved bone volume, trabecular number and trabecular thickness compared to the Sham and MM groups. Treatment with PTH also improved all of the cancellous bone parameters compared to the Sham and MM groups (Fig. 3).

### μCT evaluation of cancellous bone at the medial tibial epiphysis

**Fig. 4:**
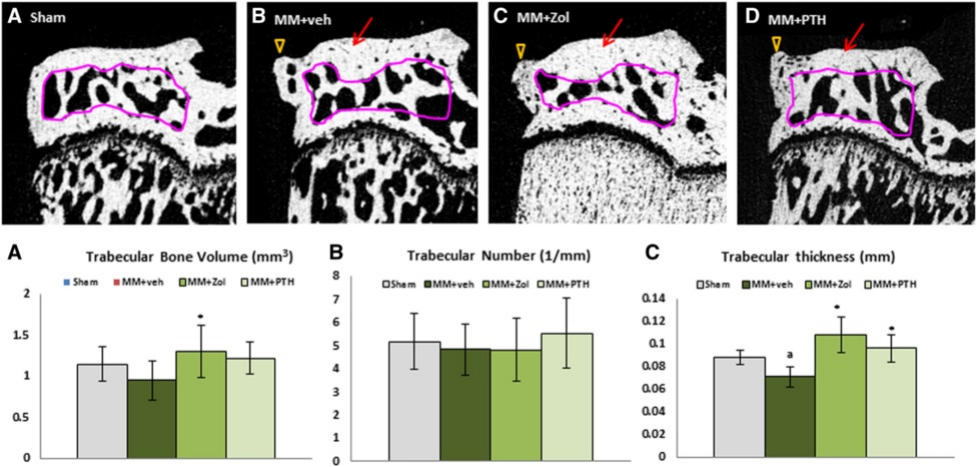
Two-dimensional micro-computed tomography (μCT) images of the medial tibial epiphysis from rats assign to study group 4A to 4D. *Pink lines* indicate area of the cancellous bone assessment. *Arrowheads* indicate osteophytes; *arrows* indicate accumulation of subchondral bone. Graph underneath depicts trabecular bone volume (**a**), trabecular number (**b**) and trabecular thickness (**c**) parameter obtained by μCT at proximal tibial metaphysis of the operated right leg. ^a^ *p* < 0.05 relative to sham control (Sham) rats; ^*^ *p* < 0.05 relative to medial meniscectomy vehicle-treated (MM + veh) rats

Local change in mechanical loading following the MM surgery resulted in different morphology of the medial epiphysis due to thickening of subchondral cortical bone and formation of osteophytes (Fig. 4). As a result the cancellous bone volume was either moderately decreased (MM + veh) or increased (MM + Zol and MM + PTH). Treatment with Zol improved cancellous bone volume largely through increase in trabecular thickness (*p* < 0.05). Trabecular number and trabecular thickness (*p* < 0.05) were both increased in PTH-treated rats compared to the MM + veh controls (Fig. 4).

### EPIC μCT evaluation of the articular cartilage at the medial tibial plateau

**Fig. 5:**
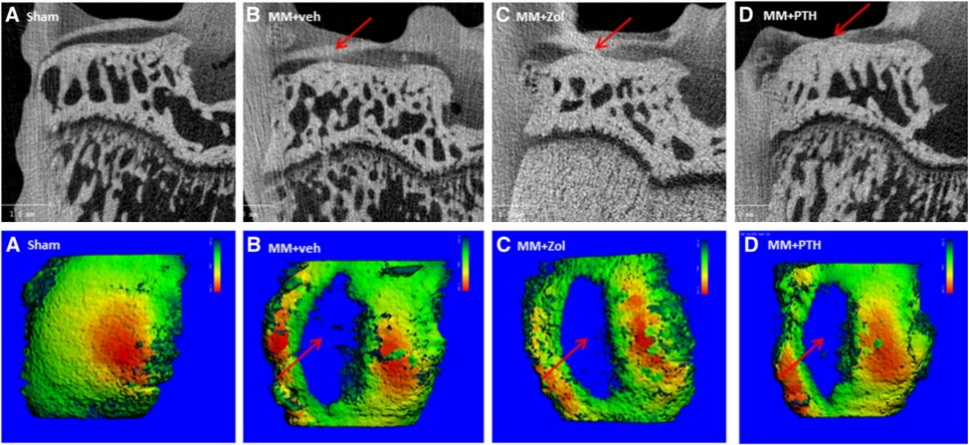
Top row shows EPIC micro-computed tomography (μCT) images of the articular cartilage covering the medial tibial plateau from rats assign to study group 5A to 5D. *Arrows* indicate cartilage defects present in all three groups of operated rats. Second row depicts large cartilage defects extending over the lateral half of Zone 1 and medial half of Zone 2, as indicated by *arrows* in three-dimensional μCT color thickness (“heat”) map of the articular cartilage at the medial tibial plateau

The cartilage volume was significantly greater in Zones 1 and 3 (Z1 and Z3) but was significantly lower in Zone 2 (Z2) in all of the MM rats compared to the sham controls. The cartilage volume values were similar in all three groups of MM rats. The cartilage was significantly thicker in Z1 in all of the MM rats compared to the controls, but it was considerably thinner in Z2 in the MM rats compared to the sham controls. The cartilage thicknesses were similar in Z3 in all of the study groups, although the values were somewhat smaller in the MM rats than in the controls (Table 2, Fig. 5).

**Table 2:** Zonal measurement of the cartilage volume and thickness at the medial tibial plateau

				S T U D Y G R O U P S		
Parameter	Unit	Zone	Sham	MM + veh	MM + Zol	MM + PTH
		z_1_	0.12 ± 0.02	0.19 ± 0.06^*^	0.19 ± 0.06^*^	0.19 ± 0.06^*^
Cartilage	mm^3^	z_2_	0.20 ± 0.02	0.11 ± 0.04^**^	0.12 ± 0.03^**^	0.12 ± 0.06^**^
Volume		z_3_	0.19 ± 0.04	0.23 ± 0.04^*^	0.23 ± 0.05^*^	0.23 ± 0.06^*^
		z_1_	0.14 ± 0.02	0.18 ± 0.06^*^	0.18 ± 0.04^*^	0.18 ± 0.07^*^
Cartilage	mm	z_2_	0.23 ± 0.02	0.15 ± 0.04^**^	0.15 ± 0.03^**^	0.15 ± 0.06^**^
Thickness		z_3_	0.23 ± 0.04	0.20 ± 0.03	0.21 ± 0.03	0.20 ± 0.05

*Sham*, sham control group, *MM + veh* medial meniscectomy (MM) vehicle-treated group, *MM + Zol* MM + zoledronate group, *MM + PTH* MM *+* parathyroid hormone group

^*^ *p* < 0.05 or ^**^ *p* < 0.001 relative to sham rats

### Histology

**Fig. 6:**
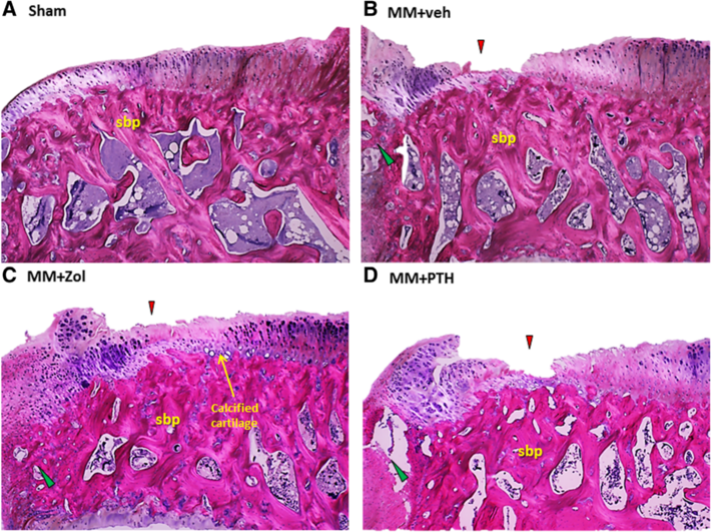
Polarized light picture of the proximal tibial epiphysis stained with hematoxylin and eosin (H&E) from rats assign to study group 6A to 6D. All three groups of medial meniscectomy (MM) rats had thicker subchondral bone plate (sbp), particularly the rats treated with zoledronate (Zol). Additionally, the calcified cartilage layer seemed to be better preserved in the Zol-treated group. The *arrowhead* indicates defects of the articular cartilage, and the *green arrowhead* indicates osteophytes

**Fig. 7:**
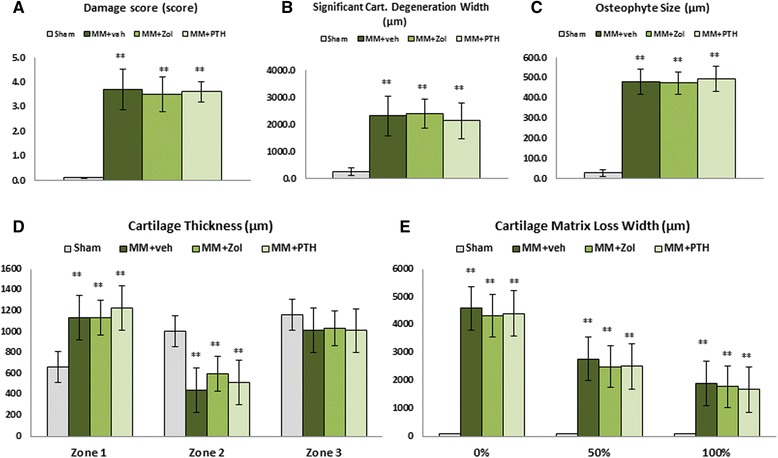
The grading of osteoarthritis (OA) lesions using damage score (**a**), significant cartilage degeneration width (**b**), osteophyte size (**c**), cartilage thickness (**d**), and cartilage matrix loss width (**e**). ^**^Significantly different from sham control group (Sham) at *p* < 0.01

Histological evaluation of the articular cartilage revealed classic images of cartilage degradation caused by the MM surgery, including a thinning to complete absence of the cartilage due to chondrocyte death or atrophy, cartilage fibrillation and the presence of osteophytes. In the control rats, the articular cartilage at the medial tibial plateau grew progressively thicker from the most medial (Z1) to the most lateral part (Z3), as revealed in histological sections and scoring (Fig. 6a; 7d). As a result of mechanical imbalance caused by the MM surgery, articular cartilage at the most medial half of Z1 next to the osteophytes was thickening, whereas the most lateral half of Z1 became very thin. The cartilage was very thin and in some cases completely missing in Z2 in the MM + veh rats, whereas the cartilage thickness in Z3 was comparable between the Sham and MM rats (Fig. 6b; 7a-d). Treatment with Zol and PTH did not have significant effects on the thickness of the articular cartilage or on the formation and size of the osteophytes (Fig. 6c and d; 7a-d).

### Dynamic histomorphometry

**Fig. 8:**
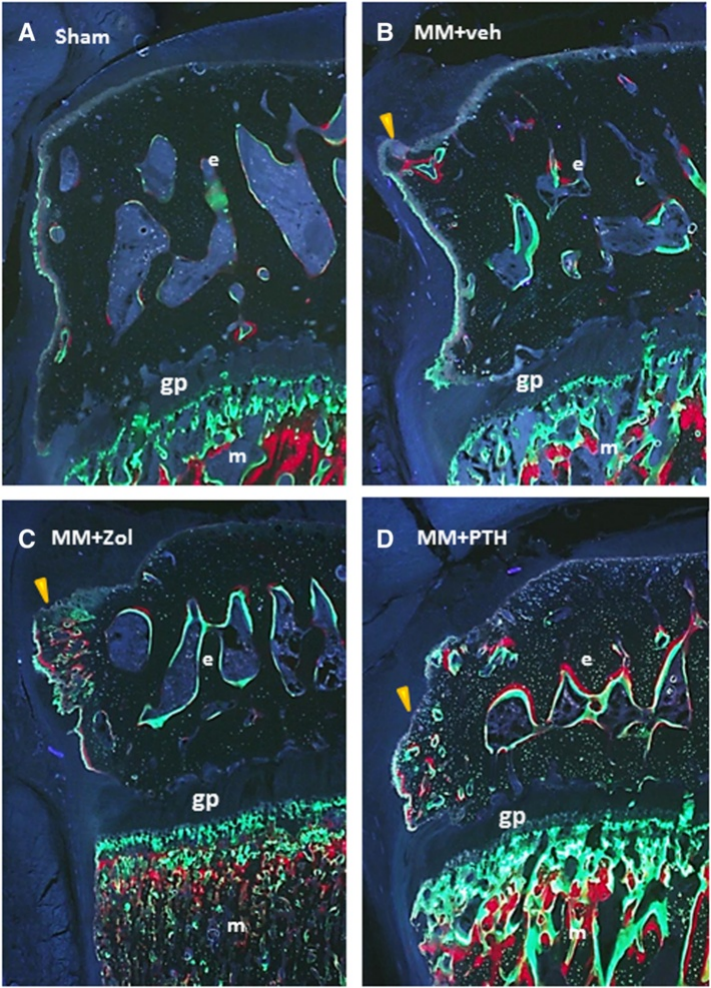
Ultraviolet (UV) images of the medial tibial epiphysis. There is little bone formation in the epiphysis of the sham control (Sham) (**a**) rat and more intensive bone formation and buildup of the subchondral bone and osteophyte formation (*arrow*) in the epiphysis of the medial meniscectomy vehicle-treated (MM + veh) rat (**b**). Intensive formation of subchondral bone plate and osteophyte formation is seen in the MM + zoledronic acid (Zol)- (**c**) and MM + parathyroid hormone (PTH)-treated rats (**d**); however, more intensive labeling with both alizarin red and osteocalcin is evident in the PTH-treated rats. The *arrowheads* indicate osteophyte formation; *e* – epiphysis; *m* – metaphysis; *gp* – growth plate

Labeling of actively mineralizing bone surfaces with calcein and alizarin red revealed active bone remodeling of the epiphyseal bone and osteophytes in all of the MM rats. Treatment with Zol and PTH did not influence the size of the osteophyte formation; however, bone remodeling was more active in the epiphysis of Zol- and PTH-treated rats than in the Sham and MM + veh controls (Fig. 8).

## Discussion

Osteoarthritis is the most common form of arthritis in the United States and is a leading cause of disability [2, 3]. The incidence and prevalence of OA is rising due to an aging population and the growing frequency of risk factors including obesity and genetics, as well as joint-specific factors that are likely reflective of abnormal loading of the joints [48, 49]. In studying OA, several methodological challenges exist that can hamper our ability to identify pertinent relationships. OA is typically defined by radiographic findings and concurrent symptoms.

Modes of locomotion and limb use differ among species [50, 51], but the ability of the mammalian skeleton to adapt to mechanical needs has long been recognized and has been demonstrated in a variety of experimental models [52–55]. In contrast to bipeds, which can only shift body weight loads to contralateral limbs, quadrupeds have the capacity to transfer and disperse loads between the front and hind legs. The DWB data from this study revealed that the sham control rats steadily increased body weight loads on the front and rear legs in proportion to age-related weight gain. Joint instability and associated pain prevented the MM rats from increasing the weight-bearing load on the operated right leg; instead, the shift in weight load toward the front legs occurred much earlier in the MM rats than in the sham controls. Although the physical activity of laboratory rats is rather limited, shifts in weight bearing toward the front legs and deloading of the right hind leg in MM rats are likely natural attempts of injured animals to prevent further joint damage and to reduce pain. Therefore, similar to the disabilities observed in OA patients, the pain associated with cartilage and bone pathology in MM rats resulted in an inability of the injured leg to perform routine mechanical functions. As a consequence of reduced weight bearing, the MM control rats showed a loss of cancellous bone at the proximal tibial metaphysis in the operated leg. Aggressive treatment with Zol and PTH resulted in significant gains in cancellous bone by increasing both the trabecular number and trabecular thickness, albeit via different mechanisms [56, 57], but both treatments failed to improve the weight-bearing capacity of the operated right leg in the MM rats.

Clinical studies in OA patients have shown limited diagnostic and prognostic value of bone turnover biomarkers [58, 59]. The stages of the disease and assay variability limit the wider use of biomarkers, although combinations of several biomarkers seem to improve their prognostic value in clinical use [60, 61]. Although both sham control and MM rats showed an increase in osteocalcin and CTX-I and decrease in P1NP and CTX-II between week 5 and week 10, elevated CTX-II values indicate higher cartilage degradation in MM rats relative to sham controls. Observed decrease in bone formation (osteocalcin, P1NP) and cartilage degradation (CTX-II) was quite expected in Zol-treated rats [14, 62–65], however, the lack of effect on bone resorption marker CTX-I was rather unexpected, in particular since the high dose of Zol was used and histomorphometry and μCT data showed clear gain in bone mass and accumulation of primary spongiosa in the metaphyses of Zol-treated rats. Bone resorption and cartilage degradation biomarkers seem to be correlated strongly in OA patients, although the origin of CTX-II has been questioned [66–68]. In PTH-treated rats there was an increase in serum osteocalcin (*p* < 0.01) and P1NP, and decrease in CTX-II values at the 5-week time point, but at the end of study serum biomarkers in PTH and MM groups were similar. Again, based on histomorphometry and μCT data we expected to see an increase in P1NP and perhaps osteocalcin throughout the study, or eventually to see an increase in bone turnover due to the high and continuous dosing with PTH.

Interpretation of changes in serum biomarkers over time is not always easy, particularly in rats, due to the ongoing modeling and remodeling of bone and cartilage [69, 70]. Clearly, some degree of discrepancy between serum biomarker data and μCT and histology findings is to be expected because serum biomarkers reflect the bone or cartilage metabolism of the entire skeleton for the particular time point when the serum was collected, whereas the focus of imaging is the very precise site of the cartilage and bone lesion that accumulates over a period of time. This difference is particularly evident in OA because different parts of the skeleton or even different areas within the same bone experience reverse metabolism, e.g., subchondral bone undergoes osteosclerosis and forms osteophytes while cancellous bone at the metaphysis is being resorbed. Similarly, articular cartilage around osteophytes in Zone 1 experience hypertrophy, while the cartilage in the Zone 2 is undergoing degradation. The biomarker results from this study support the notion of Karsdal and colleagues [71], who suggested that the interpretation of biomarkers is not always straightforward because several variables, including technical issues with assays, the stage of the disease and the treatment paradigm, can influence the significance of the data.

The subchondral bone consists of the subchondral bone plate (cortical layer) and the subarticular spongiosa (trabecular bone) and is separated from the calcified zone of the articular cartilage by the cement line [4, 5]. The articular cartilage and subchondral bone plate exhibit considerable topographical variations in thickness, density, biochemical composition and vascularity as a consequence of the different mechanical loads that exist within the same joint. Greater bone density and strength are regularly found in the more heavily loaded regions of the joint surface [72–74]. It was postulated, therefore, that thicker articular cartilage is needed in the regions of the joint with low congruency to deform, absorb and distribute more easily the load-bearing stress on the subchondral bone [75, 76]. The thickening of the articular cartilage in the most medial part of Zone 1 observed in the MMT rats certainly supported this notion. Consequently, the subchondral bone must be sufficiently strong to absorb and further distribute weight-bearing loads, yet it must contain marrow space, blood vessels and nerves to enable bone remodeling and to supply nutrients and oxygen to the subchondral bone plate and calcified cartilage [75, 77–79]. Eventually, the subchondral bone plate becomes too thick or sclerotic with the progression of OA, and when the subchondral bone plate becomes exposed after the complete loss of the articular cartilage, the chondrocytes in the calcified cartilage become dependent on the perfusion of the synovial fluid as the only source of oxygen and nutrients [80]. Unwarranted thickening of the subchondral bone could also impact the mineralization front called “tidemark” to fulfill its biomechanical role when transferring loads from uncalcified cartilage to calcified cartilage and the subchondral bone plate [81, 82].

Due to the close interaction and interplay between the articular cartilage and subchondral bone plate, it is reasonable to hypothesize that antiresorptive drugs might also reduce cartilage degradation, inhibit excessive turnover of the subchondral bone [14–18] and prevent osteophyte formation [14, 62], or that bone anabolic drugs could also stimulate cartilage synthesis by chondrocytes, preserving cartilage integrity [25–27] useful in treating OA. The results of our study clearly show that partial disuse of the operated limb resulted in mild loss of trabecular bone at the metaphysis and epiphysis of the proximal tibia, and that both Zol and PTH possess the capacity to prevent cancellous bone loss and even cause an increase in trabecular thickness at both tibial sites despite reduced usage of the injured limb. However, both tested therapies failed to reduce increased remodeling of the subchondral bone and formation of the osteophytes as documented by μCT and histomorphometry, or to prevent degradation of the articular cartilage as evidenced by EPIC μCT and histology.

It was previously suggested that osteoclast-mediated resorption of mineralized cartilage at the subchondral bone-cartilage interface is an early initiating event in OA pathophysiology and that only early bisphosphonate use after OA induction could result in the observed positive effect on cartilage health [28]. Although the rats used in our study received early and aggressive treatment with Zol, the sole antiresorptive therapy failed to prevent cartilage deterioration and osteophyte formation as proposed by others [13–15, 62]. Our results, however, are in agreement with clinical results showing that antiresorptive therapy decreased biomarkers of cartilage degradation but did not decrease the symptoms or slow the radiographic progression of OA [19, 83]. Our data also emphasize that the primary functional determinant of bone architecture is derived from the bone cells’ adaptive responses to their recent loading history. Thus within the same bone, different bone compartments can experience modeling-driven bone formation (osteophyte formation), thickening of the subchondral bone due to increased bone formation and increased transmitted force on to the underlying bone, or bone loss at the metaphysis due to disuse of the injured limb.

In surgical models of OA that mimic the development of posttraumatic OA, the cartilage damage caused by the surgery precedes changes in the subchondral bone. It is thus readily understandable that cartilage damage and pain lead to mechanical imbalances caused by weight shifting, incongruent joint surfaces and disuse causing changes (thickening) to the subchondral bone plate and osteophyte formation. Therefore, it should be borne in mind that although surgical preclinical models are good models for posttraumatic OA, they do not necessarily represent the more complex etiology of OA occurring in humans. Translational research, including animal models and biomarkers, is a critical step toward understanding and mitigating the long-term effects of the disease process as well as developing therapeutic interventions that would likely target various mechanisms to reduce joint inflammation, improve cartilage regeneration and recovery, curb bone remodeling, reduce disuse caused by pain and improve joint flexibility and recovery.

## Conclusions

The various methods utilized in this study showed that aggressive treatment with Zol and PTH does not have the capacity to prevent or correct the deterioration of the hyaline cartilage, thickening of the subchondral bone plate, osteophyte formation and mechanical incapacity of the osteoarthritic knee. It seems unlikely that a single drug will have the capacity to reduce joint inflammation, curb excessive bone remodeling, improve cartilage regeneration and reduce pain and that a multitherapeutic approach is warranted to treat posttraumatic OA.

## Abbreviations

CTX-I: cross-linked type I collagen C-terminal telopeptide
CTX-II: C-terminal telopeptide of type II collagen
DWB: dynamic weight bearing
IACUC: Institutional Animal Care and Use Committee
MCL: medial collateral ligament
MM: medial meniscectomy
OA: osteoarthritis
OARSI: Osteoarthritis Research Society International
OP: osteoporosis
P1NP: procollagen type 1 N-terminal propeptide
PTH: parathyroid hormone
PTOA: posttraumatic osteoarthritis
ROI: region of interest
sc: subcutaneous
US NIH: United States National Institutes of Health
Z1: Zone 1
Z2: Zone 2
Z3: Zone 3
Zol: zoledronic acid
μCT: micro-computed tomography
